# Knowledge and Associated Factors of Medical Students Regarding Radiation Exposure from Common Diagnostic Imaging Procedures at the University of Gondar, Ethiopia

**DOI:** 10.4314/ejhs.v30i4.14

**Published:** 2020-07-01

**Authors:** Dagnachew Eyachew Amare1, Henok Dagne1

**Affiliations:** 1 Department of Environmental and Occupational Health & Safety, Institute of Public Health, College of Medicine and Health Sciences, University of Gondar, P.O. Box, 196, Gondar, Ethiopia

**Keywords:** Diagnostic imaging procedures, Ionizing radiation, Knowledge, Medical students, Radiation exposure

## Abstract

**Background:**

Physicians’ knowledge about radiation exposure and risks was previously reported as inadequate. Therefore, the aim of this study was to assess knowledge and associated factors regarding radiation exposure among medical students from common diagnostic imaging procedures at the University of Gondar.

**Methods:**

A cross-sectional study was conducted to assess knowledge and associated factors regarding radiation exposure among medical students. A total of 473 medical students (first through sixth years of study) completed a structured questionnaire. Univariate and multi-variable binary logistic regression was used to see the factors associated with knowledge of medical students on radiation sources, exposure and risks. Variables with p-value <0.2 during the bivariable binary logistic regression were tested in the multivariable binary logistic regression. P-value<0.05 was used to declare significant association at the final model.

**Result:**

Response rate was 100%. Two hundred fifteen (45.5% 95% confidence interval (CI )(41.0%–50.3%)) participants had good knowledge regarding radiation exposure from diagnostic imaging procedures. Only 177(37.4%) participants correctly knew that Computer Tomography (CT) use X-ray. However, subjects incorrectly named magnetic resonance imaging (MRI) as if it used x-ray (12.1%) and source of ionizing radiation (19.5%). Being female [Adjusted-odds-ratio (AOR)=1.57,95% CI(1.05,2.36)], 18-20 years of age [AOR=2.18, 95% CI(1.26, 3.76)], and 1^st^ to 3^rd^ year of study [AOR=3.64, 95% CI(2.23,5.95)] were predictors of knowledge identified.

**Conclusion:**

The results highlight that medical students need to be trained well with sufficient radiological education that enable them later to adhere to safe practices.

## Introduction

The advancement of medical technologies in healthcare has paved the way to an increased use of radiological diagnostic procedures to accurately diagnose a variety of diseases and injuries. While these technologies undoubtedly give life-saving diagnosis and treatment for patients, inappropriate utilization may lead to undesired exposures to ionizing radiation which pose a long-term risk of cancer development. Ionizing radiation is known for causing cancer (1–7). In addition to cancer, ionizing radiation is known for causing adverse health effects such as cataract (3,8), genetic mutation (5) and skin erythema (9).

Radiology departments in health institutions employ several diagnostic imaging modalities with either ionizing or non-ionizing radiations for diagnostic purposes. The most common ionizing radiations sources in healthcare are radiography, computer tomography (CT), interventional fluoroscopy, mammography, angiography, and nuclear medicine while for non-ionizing radiations are ultrasound and magnetic resonance imaging (MRI). Approximately, 15% of ionizing radiation exposure comes from medical radiation (7). The emerging of CT scanning has enabled faster imaging with accuracy and hence wider use by clinicians for diagnostic technique. However, this has led to a tremendous rise in the number of people exposed to relatively high dose ionizing radiation (10,11). A survey conducted in Britain in 1989 has revealed that children younger than 15 years of age received 4% of all CT examinations (12). However, by 1999, this figure has increased to 11.2% (13).

Even though it is assumed that there is no safe dose of ionizing radiation (14), the degree of damage caused by radiation depends on a number of factors such as dose, dose rate, type of radiation, the part of the body exposed, age and health (15,16). For example, from body parts, breast tissue, and human fetus (16) and thyroids (1,17) are particularly sensitive towards radiation. Among age groups, children are the most sensitive to radiation because in children, the lifetime cancer risks due to exposure to radiation are greater than those in the adult population (18).

There is growing concern due to the increased use of radiological imaging in healthcare practices. However, the knowledge of medical professionals including medical students regarding the radiation doses and associated risks is inadequate (19–24).

Such lack of awareness about radiation source, dose and its risks may result in a potential biological life time risk for patients, particularly relevant for children and young patients. Hence, it is extremely paramount that medical students before they become practitioners to be aware of that all patients’ exposure to radiation need to be justified and kept as low as possible.

In Ethiopia, to our knowledge, there is only one published study about radiation knowledge of medical students. It was done only on final year medical students and it does not assess factors associated with knowledge (25). Core training about radiology during education is likely to be the predominant factor as widely reported by literatures for the significant gap among medical doctors about radiation exposure and its health risks. Moreover, physicians are increasingly becoming dependent on radiology-based examination as medical technology become more advanced. Hence, medical students need to have substantial knowledge about medical radiation source, exposure and health risks before they become practitioner to safeguard patients from unreasonable exposure. This study was conducted by considering the limitation of the previous study on medical students in Ethiopia, and the absence of similar study at the University of Gondar.

Therefore, the objective of this study was to determine the level of knowledge and associated factors regarding radiation exposure from common diagnostic imaging procedures among medical students at the University of Gondar, Ethiopia.

## Methods

### Study area and design:

The study was conducted at the University of Gondar, Gondar town, Ethiopia. The total number of medical students and interns enrolled in 2018 was 1450. A cross-sectional study was conducted on 473 medical students (first to sixth years which include interns, all at their penultimate year in medical school) using a questionnaire to assess their knowledge on medical radiation sources, exposure and risks.

### Source and study population:

All medical students enrolled at the University of Gondar were the source population. The study population were medical students who were randomly chosen to fill the questionnaire. The study participants were selected using computer generated simple random sampling technique using the students’ registration logbook. However, when a student refused to participate, the next number from the list was taken. Students were allowed to refuse if they wish not to participate in the study.

The following operational definitions are used in this study:

### Knowledge:

Knowledge regarding radiation exposure from common diagnostic imaging procedures was assessed by asking respondents 57 knowledge questions with Yes/No categories. The items about knowledge of radiation exposure include information such as source of radiation exposure, dose, type, bodies’ sensitivity, risk of radiation, category of diagnostic procedures. Respondents were awarded 1 point for each right answer and 0 for wrong reply. The sum was dichotomized as good and poor using mean score since the data was normally distributed. Study subjects who scored mean and above mean of the knowledge questions were considered as having “good knowledge” whereas those participants who scored below the mean of the knowledge questions were considered as having “poor knowledge”.

### Sample size determination and sampling technique:

Single population proportion formula (26) was used to determine the sample size with proportion of study subjects with good knowledge regarding radiation = 50% as there areno other previous similar studies in Ethiopia Considering 5% non-response rate, the final allowable sample size became 404.

### Data collection tool and methods:

Data collection tool was prepared from literature survey (19,24,25,27). The questionnaire consists of two parts. The first part was about sociodemographic characteristics, and the second part was regarding knowledge about radiation exposure from common diagnostic imaging procedures.

After the study subjects were informed about the objective the study, questionnaires were distributed for participants who volunteered. Accordingly, the result of the study was based on the findings or the information obtained from the self-reported questionnaire.

### Data processing and analysis:

The collected questionnaire was coded, and data were entered to Epi Info version 7. The entered data was analyzed by using the Statistical Package for Social Sciences (SPSS) version 20.0 software. Mean, percentage and interquartile range were used for analysis of descriptive statistics. Univariate binary logistic regression was used for selecting variables with p-value less than 0.2 for multivariable binary logistic regression. Hosmer and Lemeshow goodness of fit was used to test model fitness (p>0.05). Finally, AOR and 95 % CI were used to report association. P-value less than 0.05 was used to declare statistical significance.

### Ethical considerations:

Ethical approval was obtained from the University of Gondar, College of Medicine and Health Sciences Department of Environmental and Occupational Health and Safety Ethical Review Committee.

## Results

### Sociodemographic characteristics:

Four hundred and seventy-three medical students filled the self-reported questionnaire. The mean age of the study participants was 21.73±2.2.04. More than half (56.4%) of the study subjects were females. Only 60(12.7%) had ever attended training regarding radiation (Table 1).

**Table 1:** Sociodemographic characteristics of the study subjects about knowledge regarding radiation exposure from common diagnostic imaging procedures (n=473).

Variables	category	Frequency (n)	Percent (%)
Sex	Male	267	56.4
	Female	206	43.6
Year of study	1 to 3 years	235	49.7
	4 to 6 years	238	50.3
Age	18–20	138	29.2
	21–28	335	70.8
Ever exposed to radiation diagnosis	No	313	66.2
	Yes	160	33.8
Ever taken training regarding radiation	No	413	87.3
	Yes	60	12.7

### Medical student knowledge regarding radiation exposure from common diagnostic imaging procedures:

Two hundred and fifteen (45.5%, 95% CI (41.0%–50.3%)) study subjects had good knowledge regarding radiation sources, exposure and health risks. The majority of the study subjects (67.9%) mentioned that ionizing radiation has more dangerous health risks than the non-ionizing form. One hundred eighty and nine (40%) of them responded that chest is given the greatest radiation dose compared to abdominal (24%) and head (26.8%). Two hundred and sixty-eight (56.7%) 177(37.4%) of the subjects correctly named CT, did not know about the strongest radiation while 57(12.1%) incorrectly chosen MRI. CT source from the given medical diagnosis (40.2%) was chosen as the dominant ionizing modalities. Others (15%) incorrectly named radiation source over the others. However, MRI as strongest source. When they were asked 92(19.5%) participants incorrectly labelled MRI to pick diagnosing method that use X-rays, as source of ionizing radiation (Table 2).

**Table 2:** Sample specific knowledge outcomes regarding radiation exposure from common diagnostic imaging procedures and factors associated at the University of Gondar, 2019 (n=473).

Variable		Frequency (n=473)	Percentage (%)
Knowledge about radiation		Good	215
		Poor	258
Most source of radiation and its risk education	Did not have	401	84.8
	Medical education	39	8.2
	Radiologist	22	4.7
	*Others	11	2.3
Those who said YES when asked: Sources of radiation in medical diagnosis and treatment facilities.	Mammography	242	51.2
	Angiography	148	31.3
	Radiography	364	77
	CT	308	65.1
	IVP	120	25.4
	MRI	208	44
	Barium study	131	27.7
	Interventional fluoroscopy	109	23
Which body part is the least sensitive for radiation exposure?	Thyroid	43	9.1
	Breast	30	6.3
	Kidney	75	15.9
	Don’t know	325	68.7
**Those who said YES when asked:** Diagnosing method that use **X-rays?**	Mammography	168	35.5
	Angiography	90	19
	CT	177	37.4
	IVP	88	18.6
	MRI	57	12.1
	Barium study	99	20.9
	Interventional fluoroscopy	63	13.3
**Those who said YES when asked to** indicate diagnostic procedure that has ionizing radiation source?	Mammography	99	20.9
	Angiography	54	11.4
	Radiography	180	38.1
	CT	190	40.2
	IVP	62	13.1
	MRI	92	19.5
	Barium study	66	14
	Interventional fluoroscopy	41	8.7
	Don’t know	135	28.5
Which one of the following is most sensitive to radiation?	Children	306	64.7
	Adults	35	7.4
	Old age	116	24.6
	Don’t know	74	15.6

* others means physician, friends and media (mass, social, internet)

### Factors associated with knowledge of medical student regarding radiation exposure from common diagnostic imaging procedures:

Sex, age and year of study were found to be factors associated with knowledge of medical students regarding medical radiation sources, exposure and health risks in the final multivariable binary logistic regression model. Female medical students were 1.57 times more likely to have better knowledge about radiation exposure from common diagnostic imaging procedures [AOR=1.57, 95% CI (1.05, 2.36)] as compared to males. The study participants with year of study from first through third years had 3.64 times more likely to report better knowledge [AOR=3.64, 95% CI (2.23, 5.95)] as compared to those from fourth through six years. Participants in the age of 18–20 years were 2.18 times more likely to have better knowledge [AOR=2.18, 95% CI (1.26, 3.76)] as compared to those in the age of 21–28 years (Table 3).

**Table 3:** Factors associated with knowledge regarding radiation exposure from common diagnostic imaging procedures among medical students at the University of Gondar (n=473).

Variables	Categories	Knowledge		COR (95% CI)	AOR (95% CI)
		Good (%)	Poor (%)		
Sex	Male	107(40.1)	160(59.9)	1	1
	Female	108(52.4)	98(47.6)	1.65(1.14, 2.38)	1.57(1.05,2.36)*
Year of study	1 to 3 years	154(65.5)	81(34.5)	5.52(3.71,8.20)	3.64(2.23,5.95)***
	4 to 6 years	61(25.6)	177(74.4)	1	1
Age	18–20 years	101(73.2)	37(26.8)	5.29(3.41,8.21)	2.18(1.26,3.76)***
	21–28 years	114(34)	221(66)	1	1
Ever exposed to radiation diagnosis Ever taken training regarding radiation	Yes	64(40)	96(60)	1	1
	No	151(48.2)	162(51.8)	1.40(0.95, 2.06)	-
	Yes	18(30)	42(70)	1	1
	No	197(47.7)	216(52.3)	2.13(1.19,3.82)	-

* p-value <0.05,

*** p-value <0.001, Hosmer and Lemeshow goodness of fit p-value= 0.900

## Discussion

Beside its immense benefits, radiation from medical imaging procedures such as CT is becoming a concern due to high radiation dosage and an ever-increasing usage for examination, and its associated risks of cancer. It is in particular an important issue for children as they are sensitive towards radiation and have longer lifespans. Medical students are future medical practitioners. Therefore, it is important to emphasize about adherence to safe practice in the event of radiological examinations which require substantial knowledge of ionizing and non-ionizing radiation source, exposure and health risks during their formal education. When students have better knowledge regarding radiation exposure, they can protect themselves, the patients and the patients’ caretakers from unnecessary radiation exposure, and teach the general population about radiation exposure and risks.

In this study, two hundred and fifteen (45.5%) subjects had good knowledge regarding radiation sources, exposure and health risks. Their poor performance could be related to lack of training regarding radiology as 87.3% study participants already mentioned that they did not have training. An alternative reason for this gap could be that medical instructors might undermine the need to teach radiation exposure and risks as they teach disease diagnosis procedures. Moreover, limited availability of books, pamphlets and digital materials regarding medical diagnostic procedures and radiation safety in the library may have played role for their poor knowledge regarding radiation exposure and risks. The general knowledge in the current study is greater than that of previous findings, 31.6% (28), 38% (29) and 37.8% (30). The difference in good knowledge regarding radiation exposure from common diagnostic imaging procedures among these studies could be due to the variation in the type of assessment tools used, difference in study setting, medical curricula, and radiation training in addition to difference in sociodemographic characteristics. There is no structured guideline regarding medical radiation protection education and training in Ethiopia. However, medical students get limited information regarding radiation safety from different subjects they take in the due course in unorganized manner. Consequently, developing comprehensive medical radiation protection education and training guideline and incorporating radiation safety in the curriculum in a sufficient detail is crucial for raising the knowledge of healthcare providers in protecting the health professionals, patients and the general public from unnecessary radiation exposure.

When medical students start practicing as interns or physicians, it is likely that they continue with this knowledge gap. Studies have shown that even the knowledge of physicians was poor regarding medical radiation dose, exposure and risks (20,24). As the 87.3% participants in this study disclosed, lack of training could be the reason for their poor knowledge. A three hour lecture for fourth year medical students related to medical radiation has resulted in 31% knowledge gain compared to the control group (31). A previous study showed that medical students improved their knowledge score from 59% to 70% after a lecture regarding radiation oncology (32). According to a previous study, 73.3% of medical students, where their knowledge of medical radiation score was high, indicated that medical training was the source of most of their radiation related education (27). This indicates that the poor knowledge of medical students in the current study may be mainly the result of lack of training during their education.

This study shows that 64.5%, 81%, 62.6%, and 86.7% of the study participants did not know that mammography, angiography, CT and interventional fluoroscopy use x-ray, respectively. The majority of study subjects (37.4%) correctly chose CT as the main source of x-ray followed by mammography (35.5%), while 12.1% of the participants wrongly mentioned that MRI uses X-ray. When they were asked to choose diagnostic imaging procedure that has the most radiation source, the majority of the medical students pick ‘do not know’ (56.4%), wrongly said MRI (15%) and correctly labelled CT (14.6%). Contrary to this, 86.7% of medical students at the University of Toronto in Canada accurately indicated that CT is the most source of radiation (27). The number of subjects (15%) in this study who incorrectly named MRI work with x-ray is similar with that of (29) finding where 14% of the study population answered that MRI used x-rays.

In the current study, 67.9% of the subjects replied that ionizing radiation is more dangerous than non-ionizing radiation (5.3%), while the rest mentioned ‘don’t know’ (15.3%) and ‘both are equal’ (11%). In this study, one hundred nighty (40.2%) participants knew that CT is the source of ionizing radiation, a result by far lower compared with 84% (33) and 100% of participants (27). Ninety-two (19.5%) participants in this study incorrectly labelled MRI as source of ionizing radiation. A study conducted on the final year medical students (25) in Ethiopia found that 79.3% of them wrongly pick MRI as the source of ionizing radiation which is lower knowledge score compared to the current study. It was also a better score compared with 25% (33) and 25.5% (32). However, only 3.6% of the study population in a previous study incorrectly considered MRI as a source of ionizing radiation (29) which is a greater score compared to this study.

The majority of medical the students, 306 (64.7%), correctly mentioned that children are the most sensitive for radiation exposure which is less than the result (80%) found in Canada (27) and 80% in Ireland (29). Four hundred and one (84.8%) subjects indicated that cancer is the most common health risk associated with radiation exposure followed by skin disorder (82%) and genetic disorder (79.7%). Though the majority (68.7%) did say ‘do not know’, 15.9% of the subjects correctly responded that kidney is the least sensitive towards ionizing radiation compared to thyroid (9.1%) and breast tissue (6.3%). According to a previous study, 51% of the study subjects who were exposed to radiation training and 42% of them who were non-exposed group managed to correctly identify kidney as the least sensitive body part over thyroid and breast tissues (29).

Year of study for the medical students was one of the factors associated with knowledge level. First through third years had 3.64 times more likely better knowledge than fourth through six years. It is in particular disappointing to see senior medical students including interns performed poorly to the general knowledge questions compared compared with their juniors. Similarly, McCusker et’al (33) has reported that interns (59%) had lower knowledge score regarding ionizing radiation compared with medical students (70%). Furthermore, it was reported that fourth year students (80%) were more knowledgeable regarding medical radiological question compared to final year students (55%) (34). The possible reason for this outcome could be that junior students might have high school physics knowledge that familiarizes them to those questions compared with the seniors. McCusker et’al (33) depicted that previous physics knowledge could be the reason behind better performance of juniors compared with the final year students. On the other hand, this result contradicts previous studies which demonstrated an increase in students’ knowledge of radiation with increasing year spent in medical school (25,28,29). Moreover, as the majority of the study subjects (87.3%) declared that they did not take radiation related training, spending more years in medical school might not have impact on knowledge gain on radiation dose, exposure and risk

Female subjects were more likely to have better knowledge compared with males. This finding is in agreement with a previous study which reported that females had better knowledge regarding radiation protection questions compared with males (30). This finding, however, contradicts previous findings where females scored lower level of knowledge regarding ionizing radiation compared with males for both medical students (29) and physicians (19).

Generally, inadequate knowledge of medical students about medical radiation risks may result in exposing patients to repeated high dose radiation examination when becoming a future practitioners. Year of study, female sex, and age were independent predictors of knowledge regarding radiation exposure from common diagnostic imaging procedures. Therefore, adequate radiation knowledge improving strategies such as training are required.

Finally, this study was not without limitation. This study was a self-reported study which might be affected by social desirability bias even though techniques to reduce bias have been employed. The inherent weakness of cross-sectional study that fails to establish cause and effect relationship results difficulty to identify the true determinants of knowledge in the current study.
